# New Home, New Prognosis? Reduced Hypertension Risk after Moving to a High-Walkability Neighborhood

**DOI:** 10.1289/ehp.124-A112

**Published:** 2016-06-01

**Authors:** Nancy Averett

**Affiliations:** Nancy Averett writes about science and the environment from Cincinnati, OH. Her work has been published in *Pacific Standard*, *Audubon*, *Discover*, *E/The Environmental Magazin*e, and a variety of other publications.

The impact of the built environment on health is still a fairly new research field, and many investigations have relied on the use of cross-sectional data, a research model that carries the risk of incorrectly suggesting reverse causation.^1^ A study in this issue of *EHP*, however, uses a more robust longitudinal design and reports that residents of Ontario, Canada, who moved from a low-walkability neighborhood to a high-walkability neighborhood had a 54% lower likelihood of developing hypertension than those who moved from one low-walkability neighborhood to another.^2^

“It’s a very strong study,” says Jana A. Hirsch, a postdoctoral fellow with the Carolina Population Center at the University of North Carolina at Chapel Hill. That’s especially true, she says, because there is so little research in the field, conducted in a population-based sample, that assesses a long-term outcome such as hypertension. Hirsch was not involved in the research.

**Figure:**
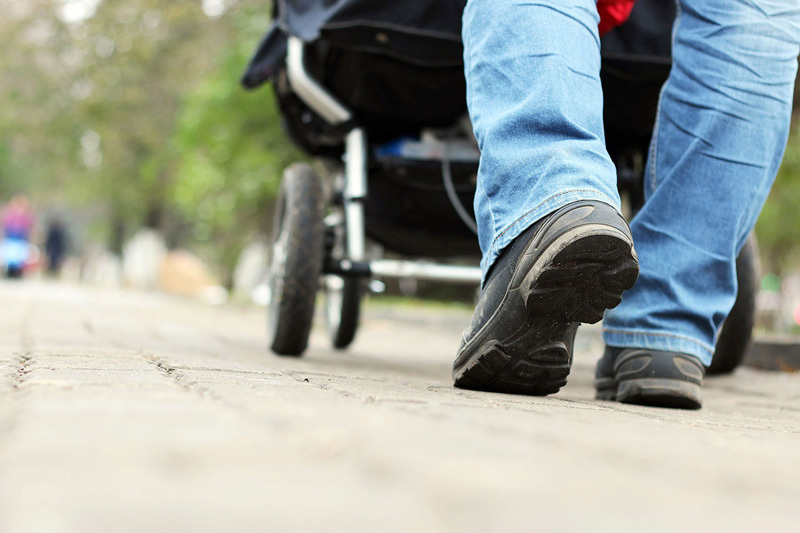
The walkability of a neighborhood is determined not just by spatial factors, such as block length, but also aesthetic factors, such as shade and availability of sidewalks. © alexkich/Shutterstock

The investigators used data from Statistics Canada’s Canadian Community Health Surveys. Participants had to be Ontario residents aged 20 years or older with a valid Ontario health card number and no previous hypertension. They also had to have moved to a new home within the study period. This was a key element of the study, because moving can be stressful, and the ability to move can be associated with both positive and negative confounders.

Neighborhood walkability was determined using Walk Score®, a proprietary measure that scores neighborhoods between 0 and 100 based on factors including block length and distance to amenities (e.g., shops, parks, and restaurants).^3^ All the participants started out in neighborhoods with a Walk Score® below 90. Those who moved to a new neighborhood with a score of 90 or higher were put in the high-walkability group, while the low-walkability group moved to a new neighborhood that also had a score below 90.

The high- and low-walkability populations differed greatly in terms of income, race, smoking rates, psychosocial stress, diet, and other factors associated with hypertension risk. To adjust for these differences, the researchers used a statistical method called propensity score matching to narrow down the two groups to individuals who were the most similar except in terms of their built environment.

“We did this so that we could compare like with like,” says lead author Maria Chiu, a scientist at the Institute for Clinical Evaluative Sciences in Toronto. “Then we could say confidently that the hypertension risk is independent of all these other factors.” The authors ended up with a cohort of 1,057 individuals in each group. Participants were followed for up to 10 years, with a median followup of 4.3 years.

Although it was necessary to set cut points in order to divide the study populations into two comparable groups, Chiu acknowledges that Walk Score® 90 was a high threshold. However, she says additional analyses conducted by the authors, which were not included in this report, showed clear dose–response relationships with cut points of 70 and 50. “So the biggest difference in hypertension rates was in [people who moved to] 90-plus [neighborhoods], less so in 70, and even less so in 50,” she says.

Hirsch agrees that it doesn’t take moving to a Walk Score® 90 neighborhood to see positive behavioral and health changes. “What if a person moves from a 0 to a 69 walkability? That’s a pretty big jump,” she says. “In the research that I’ve done,^4^ we find increases in walking and decreases in obesity for people who move to places that are just 10 points higher on Walk Score®.”

As a composite measure that combines destinations and street characteristics, Walk Score® doesn’t capture some of the aesthetic features that might affect a person’s decision to walk, such as sidewalks, shade, and snow removal. Other research has indicated that people’s perception of their neighborhood is just as important as the actual features of the neighborhood—possibly even more so.^5^

Although Walk Score® is not an easy measurement for urban planners to act upon, Chiu says the results of the study show that planners need to keep walkability in mind when they’re developing neighborhoods. That way, she says, “walking becomes the obvious choice and the more enjoyable choice rather than hopping in a car and driving to your destination.”
